# Synthesis and Characterization of High Glycolic Acid Content Poly(glycolic acid-*co*-butylene adipate-*co*-butylene terephthalate) and Poly(glycolic acid-*co*-butylene succinate) Copolymers with Improved Elasticity

**DOI:** 10.1021/acsomega.3c05932

**Published:** 2023-10-03

**Authors:** Alastair Little, Shiyue Ma, David M. Haddleton, Bowen Tan, Zhaoyang Sun, Chaoying Wan

**Affiliations:** † International Institute for Nanocomposites Manufacturing (IINM), WMG, University of Warwick, Coventry CV4 7AL, U.K.; ‡ Department of Chemistry, University of Warwick, Coventry CV4 7AL, U.K.; § PJIM Polymer Scientific Co., Ltd., Shanghai 201102, China

## Abstract

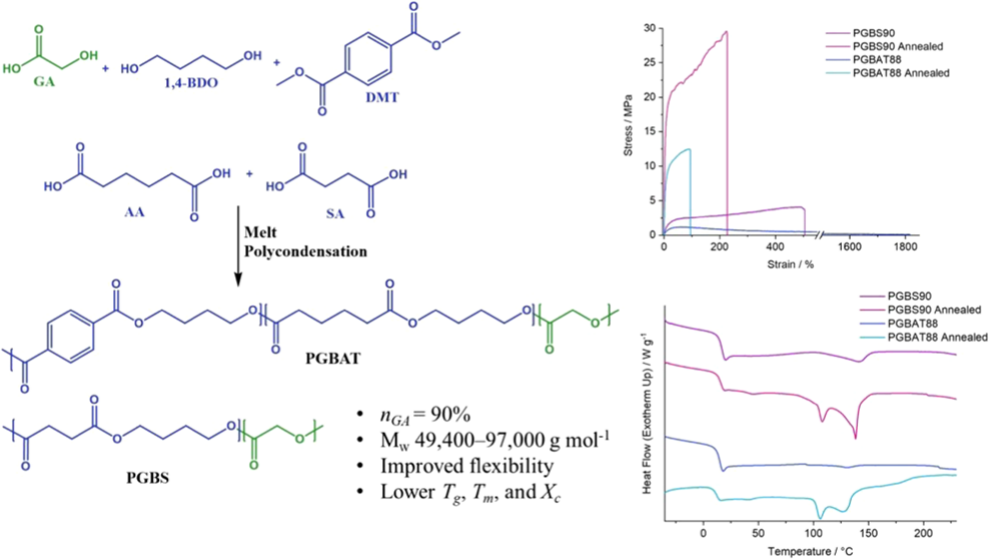

Poly(glycolic acid) (PGA) is a biodegradable polymer with high gas barrier properties, mechanical strength, and heat deflection temperature. However, PGA’s brittleness severely limits its application in packaging, creating a need to develop PGA-based copolymers with improved elasticity that maintain its barrier properties and hydrolytic degradability. In this work, a series of PGBAT (poly(glycolic acid-*co*-butylene) adipate-*co*-butylene terephthalate) copolymers containing 21–92% glycolic acid (*n*_GA_) with *M*_w_ values of 46,700–50,600 g mol^–1^ were synthesized via melt polycondensation, and the effects of altering the *n*_GA_ on PGBAT’s thermomechanical properties and hydrolysis rate were investigated. Poly(glycolic acid-*co*-butylene succinate) (PGBS) and poly(glycolic acid-*co*-butylene terephthalate) (PGBT) copolymers with high *n*_GA_ were synthesized for comparison. DSC analysis revealed that PGBAT21 (*n*_GA_ = 21%) and PGBAT92 were semicrystalline, melting between 102.8 and 163.3 °C, while PGBAT44, PGBAT86–89, PGBT80, and PGBS90 were amorphous, with *T*_g_ values from −19.0 to 23.7 °C. These high *n*_GA_ copolymers showed similar rates of hydrolysis to PGA, whereas those containing <50% GA showed almost no mass loss over the testing period. Their mechanical properties were highly dependent upon their crystallinity and improved significantly after annealing. Of the high *n*_GA_ copolymers, annealed PGBS90 (*M*_w_ 97,000 g mol^–1^) possessed excellent mechanical properties with a modulus of 588 MPa, tensile strength of 30.0 MPa, and elongation at break of 171%, a significant improvement on PGA’s elongation at break of 3%. This work demonstrates the potential of enhancing PGA’s flexibility by introducing minor amounts of low-cost diols and diacids into its synthesis.

## 1 Introduction

Widespread plastic pollution has encouraged the development of biodegradable plastics for packaging. Currently, most biodegradable plastics are limited by their poor barrier properties, low heat resistance, and slow degradation rates in natural environments. Poly(glycolic acid) (PGA) is both an established and emerging biodegradable polymer with excellent barrier properties, high tensile strength, high heat deflection temperature, and fast degradation rate that outperforms most commercial biodegradable polymers.^1−3^ PGA’s use has traditionally been restricted to biomedical applications due to its high cost. However, the recent development of large-scale, continuous PGA production processes has lowered its cost and enabled its application in packaging.^4,5^

Despite its excellent tensile strength and barrier properties, PGA’s application in packaging is hindered by its brittleness. A further problem of PGA is its susceptibility to degradation during synthesis and processing, which is partly due to its high melting point and poor thermal stability. To improve its flexibility, PGA can be blended with PBAT.^6−8^ Alternatively, copolymerization can reduce PGA’s brittleness and melting temperature. Traditionally, industrial PGA production has relied upon ROP. However, copolymerization of PGA via ROP is limited by the lack of commercially available cyclic monomers (lactide, ε-caprolactone, trimethylene carbonate, and 1,4-dioxane) and their relatively high cost. Additionally, these comonomers have low reactivity ratios compared to glycolide, leading to increased reaction times and temperatures.^9−11^ Comparatively, a wide variety of low-cost diols and diacids are available, which could be copolymerized with glycolic acid via polycondensation, allowing for the tailoring of the copolymer’s structure.

Various researchers have explored methods of polymerizing hydroxy acids with diols and diacids, such as ethylene glycol and terephthalic acid. Olewnik et al. produced a low *M*_n_ (2120 g mol^–1^) PGET (poly(glycolic acid-*co*-ethylene terephthalate)) copolymer containing 36% glycolic acid with a *T*_*m*_ of 166 °C via the melt polycondensation of bis(2-hydroxyethyl) terephthalate and glycolic acid oligomers using Sb_2_O_3_.^12^ Zhou et al. prepared PLET with *M*_w_ values of 22,600–56,000 g mol^–1^ through a one-pot ROP polycondensation method from lactide, ethylene glycol, and terephthalic acid using Sn(Oct)_2_ and a titanium catalyst.^13^ These PLETs contained 10–50% lactic acid and displayed decreases in the *T*_g_, *T*_m_, crystallinity, and tensile strength as the lactic acid content was increased. Nakayama et al. synthesized alternating PGEGT poly(ethylene diglycolate terephthalate) from the polycondensation of ethylene diglycolate and terephthaloyl dichloride at 0 °C in a tetrachloroethane and pyridine mixture. This copolymer’s molecular weight was low (*M*_n_ 8,100 g mol^–1^), but it showed increased *T*_g_ (48 °C) and decreased *T*_m_ (209 °C) compared to PGA.^14^

Hot melt adhesives containing approximately 85 wt % of glycolic acid were synthesized from glycolic acid, adipic acid, and ethylene glycol.^15^ Minor amounts of pentaerythritol and trimethylolethane were incorporated to introduce branching and achieve firmer materials. High-molecular-weight (*M*_w_ 200,000 g mol^–1^) copolymers of PLA and PBAT have also been produced by reacting a PBAT prepolymer and a PLA prepolymer together in a vacuum reactor.^16^

Recently, a process for improving the marine biodegradability of PBS, PBAT, PET, and PBT via copolymerization with 10–30 mol % of glycolic acid was disclosed.^17^ These copolymers possessed high molecular weights (*M*_n_ 48,900–56,800 g mol^–1^) and excellent mechanical properties (tensile strength 35–45 MPa, elongation at break 120–800%). These were prepared either via the polycondensation of two oligomers or the direct esterification and polycondensation of glycolic acid, diacids, and diols using tin and titanium catalysts.

Following this, poly(glycolic acid-*co*-butylene succinate) (PGBS), poly(glycolic acid-*co*-butylene terephthalate) (PGBT), poly(glycolic acid-*co*-ethylene terephthalate) (PGET), poly(glycolic acid-*co*-butylene adipate-*co*-butylene terephthalate) (PGBAT), and poly(glycolic acid-*co*-butylene furanoate) (PGBF) containing 0–60% glycolic acid have been synthesized and examined in detail.^18−23^ They all showed good mechanical properties and were semicrystalline at low glycolic acid percentages but became amorphous as the glycolic acid content increased above 30 mol %. Poly(lactic acid-*co*-butylene furanoate) (PLBF), poly(lactic acid-*co*-butylene succinate) (PLBS), poly(caprolactone-*co*-butylene furanoate) (PCBF), and poly(caprolactone-*co*-butylene succinate) (PCBS) have also been reported and displayed similar trends as the copolymer ratio was adjusted.^24−27^

**Scheme 1:**
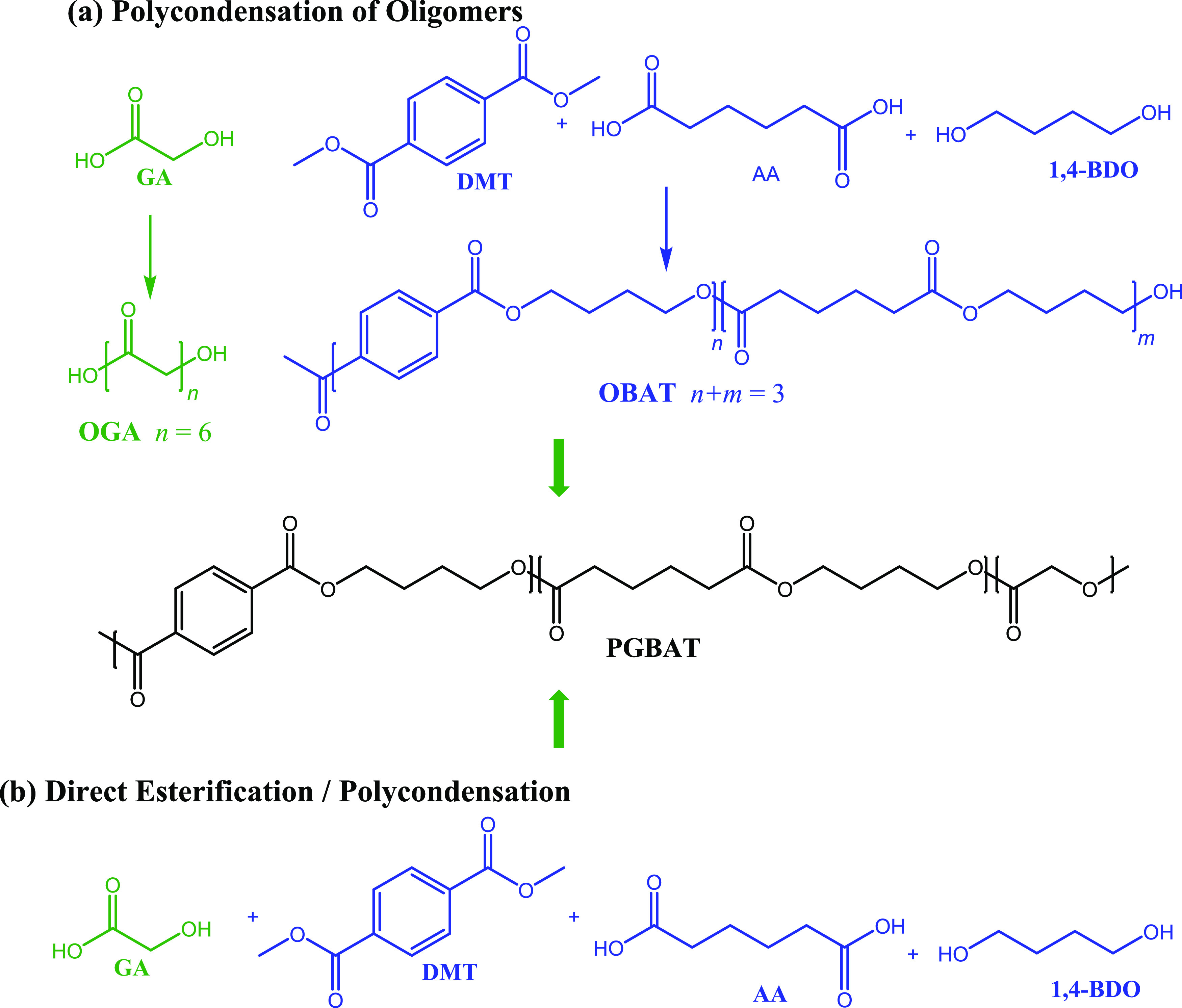
Synthesis of PGBAT via (a) Polycondensation of Oligomers and (b) Direct Esterification/Polycondensation of Monomers

Most of these researchers aimed to incorporate glycolic acid into other polyesters to improve their marine biodegradability, and while these materials display high molecular weights and good thermomechanical properties, PGA is not their major component. Having a high glycolic acid content is crucial to maintaining PGA’s excellent barrier properties, high crystallinity, and strength.^28^ Currently, there are no reports on the properties of copolymers of glycolic acid, diols, and diacids containing high glycolic acid contents. Therefore, we explored the synthesis and properties of copolymers of glycolic acid, diols, and diacids via polycondensation, where glycolic acid is the major component. Due to the excellent properties of PGA/PBAT blends, PGBAT copolymers were focused upon.^6,7^ To begin, the reaction conditions were explored using Scheme 1a,b. Then, after investigating how PGBAT’s properties varied as the content of glycolic acid was increased, PGBT and PGBS copolymers containing high glycolic acid contents were studied. Finally, the effects of annealing on the thermomechanical properties and hydrolytic stability of PGBAT and PGBS containing 90 mol % of glycolic acid were examined.

## 2 Experimental Section

### 2.1 Materials

Glycolic acid, dimethyl terephthalate, adipic acid, succinic acid, bismuth(III) subsalicylate, antimony(III) oxide, zinc acetylacetonate, zinc acetate, methanesulfonic acid, phosphoric acid, DMSO-*d*_*6*_, and CDCl_3_ were purchased from Sigma-Aldrich. 1,4-Butanediol, tin(II) 2-ethylhexanoate, and titanium butoxide were purchased from VWR, Alfa Aesar. Zirconium(IV) acetylacetonate was purchased from Strem Chemicals U.K., Ltd. Irgafos 126, Irgafos 168, and Joncryl ADR 4468 were from BASF.

### 2.2 Synthesis

#### 2.2.1 Synthesis of PGA Oligomer (OGA)

Glycolic acid (42.59 g, 0.56 mol), Sn(Oct)_2_ (363 μL, 0.2 mol %), and a magnetic stir bar were added to a 250 mL single-neck round-bottom flask attached to a vacuum distillation setup. The mixture was purged with N_2_ three times and heated at 160 °C with stirring for 6–8 h until the reaction mixture solidified. *M*_n_ was calculated as 494.8 g mol^–1^ via end-group analysis from the ^1^H NMR spectra (DMSO-*d*_*6*_, 100 °C).

#### 2.2.2 Synthesis of PBAT Oligomer (OBAT)

Dimethyl terephthalate (10.19 g, 0.052 mol), 1,4-butanediol (11.82 g, 0.13 mol), Ti(OBu)_4_ (36 μL, 0.1 mol % relative to dimethyl terephthalate and adipic acid), and a magnetic stir bar were added to a 250 mL single-neck round-bottom flask attached to a vacuum distillation setup. The mixture was purged with nitrogen three times and stirred at 160 °C for 1 h. Adipic acid (7.671 g, 0.052 mol) was then added, and the mixture was stirred at 180 °C for 4 h. *M*_n_ was calculated as 647.6 g mol^–1^ via end-group analysis from the ^1^H NMR spectra (CDCl_3_).

#### 2.2.3 Synthesis of PGBAT from Oligomers

Using PGBAT80 as an example, we added OGA (15.83 g, 0.032 mol), OBAT (5.18 g, 0.008 mol), Sb_2_O_3_ (31.5 mg, 0.15 wt %), and Irgafos 126 (42.0 mg, 0.2 wt %) to a single-neck round-bottom flask attached to a vacuum distillation setup. The mixture was heated to 190 °C and stirred under nitrogen for 30 min. The pressure was then gradually reduced to 30 mbar over 30 min. After a further 30 min, the pressure was further reduced to ≤0.1 mbar and left for 4–5 h. The reaction was considered finished once the magnetic stirrer bar could no longer stir the mixture. The molten polymer was removed and analyzed without further purification.

#### 2.2.4 Synthesis of PGBAT, PGBT, and PGBS from Monomers

Using PGBAT90 as an example, dimethyl terephthalate (2.91 g, 0.015 mol), 1,4-butanediol, (1.42 g, 0.0158 mol), Ti(OBu)_4_ (5 μL, 0.05 mol % of DMT + AA), and a magnetic stirrer bar were added to a 250 mL single-neck round-bottom flask attached to a vacuum distillation setup. The mixture was purged with nitrogen three times and stirred at 170 °C for 30 min (until the mixture solidified). Then, adipic acid (2.19 g, 0.015 mol), 1,4-butanediol (1.42 g, 0.0158 mol), glycolic acid (20.53 g, 0.27 mol), and Sn(Oct)_2_ (44 μL, 0.05 mol % of GA) were added, and the mixture was heated at 180–210 °C for 4 hours, with the temperature being increased at 10 °C per hour. After this, Sb_2_O_3_ (0.1 wt %) and Irgafos 126 (0.2 wt %) were added, and the pressure was gradually reduced to 30 mbar over 30 min. After a further 30 min, the pressure was further reduced to ≤0.1 mbar and left for 4–5 h. The reaction was considered finished once the magnetic stirrer bar could no longer stir the mixture. The molten polymer was removed and analyzed without further purification.

Copolymers synthesized for the annealing experiments in section 4.2.3 were synthesized using twice this scale (∼60 g) and a mechanical stirrer; roughly 30–35 g of polymer was obtained. Polymer yield was calculated on the basis of the mass of polymer collected and the mass of sublimed glycolide.

### 2.3 Characterization

#### 2.3.1 SEC_CHCl3_

SEC of copolymers containing *n*_GA_ ≤ 50 mol % was performed using CHCl_3_ as the eluent. SEC was carried out using an Agilent Infinity II 1260 MDS instrument equipped with differential refractive index (DRI), viscometer (VS), dual-angle light scatter (LS), and multiple wavelength UV detectors. The system was equipped with 2× PLgel Mixed C columns (300 mm × 7.5 mm) and a PLgel 5 μm guard column. The eluent was CHCl_3_ run at 1 mL/min at 30 °C. Poly(methyl methacrylate) standards (Agilent EasiVials) were used to create a third-order calibration between 1,020,000 and 1,840 g mol^–1^. Analyte samples were filtered through a 0.22 μm nylon membrane before injection.

#### 2.3.2 SEC_DMF_

SEC of copolymers containing *n*_GA_ ≥ 50 mol % was performed using DMF as the eluent. SEC was carried out using an Agilent Infinity II 1260 MDS instrument equipped with differential refractive index (DRI), viscometry (VS), dual-angle light scatter (LS), and multiple wavelength UV detectors. The system was equipped with 2× PLgel Mixed D columns (300 mm × 7.5 mm) and a PLgel 5 μm guard column. The eluent was DMF containing 5 mmol of NH_4_BF_4_ run at 1 mL/min at 60 °C. Poly(methyl methacrylate) standards (Agilent EasiVials) were used to create a third-order calibration between 1,020,000 and 1,840 g mol^–1^. Analyte samples were filtered through a 0.22 μm nylon membrane before injection.

#### 2.3.3 ^1^H NMR Spectroscopy

The *n*_GA_ value was determined via ^1^H NMR spectroscopy. NMR spectra were obtained with a Bruker Avance III HD 400 MHz Spectrometer. Copolymers containing *n*_GA_ ≤ 50 mol % were analyzed using CDCl_3_ as a solvent, and those with *n*_GA_ ≥ 50 mol % were analyzed using d_6_-DMSO. *n*_GA_ values were calculated using
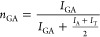
where *I*_GA_ = Integration of glycolic acid units, *I*_A_ = intergradation of adipic acid units, and *I*_T_ = integration of terephthalic acid units (see Figures S6 and S7).

DSC experiments were performed using a Mettler-Toledo DSC 1 with 5–10 mg of sample in a 40 μL aluminum DSC pan. Samples were scanned from −40 to 230 °C under an N_2_ flow at a heating rate of 10 °C min^–1^.

TGA experiments were performed using a Mettler-Toledo TGA 1 instrument with ∼10 mg of sample in an alumina crucible. Samples were scanned from 25 to 500 °C under an N_2_ flow at a heating rate of 10 °C min^–1^.

#### 2.3.4 Mechanical Tensile Testing

0.5 mm polymer films were prepared by hot-pressing at 100–190 °C. Samples were held at the same temperature without pressure for 2 min to soften the polymer and then pressed at 40 kN for 3 min. PGBAT21–44 were hot-pressed at 150 °C; PGBAT86–89, PGBT80, and PGBS90–93 were hot-pressed at 100 °C; and PGBAT92 was hot-pressed at 190 °C. The films were cut according to DIN 53504S2. Tensile testing was performed by using a Shimadzu Autograph AGS-X tester. The extension rate was set to 10 mm min^–1^ with a 10 kN load cell, and the room temperature was 21 °C. The gauge length was set to 40.4 mm. Five tensile tests were performed for each sample, and the mean and standard deviation were reported.

#### 2.3.5 Degradation Testing

0.5 cm × 1 cm pieces were then cut and immersed in 1 mL of pH 7 phosphate-buffered saline (PBS) solution at 60 °C. After degradation, each sample was washed with distilled water and dried overnight at 30 °C. Each sample was tested in triplicate.

## 3 Results and Discussion

### 3.1 PGBAT Synthesis Optimization

The synthetic procedures of PGBAT copolymers were investigated and optimized first. In a previous study reported by Han et al., PGBF copolymers were synthesized via the melt polycondensation of a glycolic acid oligomer (OGA) and a butylene furanoate oligomer (OBF) at 190–210 °C using 0.15 wt % of Sb_2_O_3_.^29^ Using a similar method (Scheme 1a), PGBAT50 was synthesized; this ratio was selected to ensure the final copolymer could be easily dissolved in common organic solvents.

While Sb_2_O_3_ is an effective polycondensation catalyst, it is a known carcinogen. Therefore, a catalyst screen was performed to identify whether an alternative could be used. However, the results (Table S1 and Figure S1) found that only Sb_2_O_3_ yielded a sufficiently high *M*_w_ (34,900 g mol^–1^). Catalyst screening was also carried out to examine the effect of glycolic acid esterification on the polycondensation of PGBAT, and Sn(Oct)_2_ was found to yield the highest *M*_w_ value (Table S2 and Figure S2). However, while these catalysts yielded high molecular weights, the resulting PGBATs were dark brown. Antioxidants were screened for their ability to reduce this discoloration, and Irgafos 126 was found to be the most effective (Table S3 and Figure S3). It reduced dispersity and *M*_w_ values but not *M*_n_, suggesting it prevents radical-induced chain branching reactions, which may lead to the high *M*_w_ values observed without an antioxidant.

Initial work (Table S4) found that when PGBATs with high *n*_GA_ values were synthesized via the polycondensation of oligomers, polymer yields were low, due to the formation and sublimation of glycolide throughout the reaction. Additionally, these copolymers displayed poor thermomechanical properties (Table S5). The synthesis of PGBAT via the direct esterification and polycondensation of monomers proved to be an easier and more effective method, resulting in higher yields and less glycolide formation, meaning that the PGBAT’s *n*_GA_ was close to its feed value (Table S6). It was also found that for high *n*_GA_ PGBAT, a lower excess of 1,4-butanediol to adipic acid and dimethyl terephthalate (B:A + T) was required to yield high *M*_w_ values (Table S7). For a feed value of *n*_GA_ = 90%, reducing the excess of 1,4-butanediol from 1.25:1 to 1.05:1 increased the PGBAT’s B:A+T ratio from 1:0.83 to 1:1 and increased the *M*_n_ from 16,100 to 21,700 g mol^–1^ (Table S7).

### 3.2 Thermomechanical Properties and Hydrolysis of PGBAT, PGBS, and PGBT

Following these studies, the effects of increasing the glycolic acid content on the thermomechanical properties and hydrolysis rate of PGBAT were investigated. Table 1 shows the molecular properties of the PGBATs synthesized. Each reaction was performed until the magnetic stirrer bar could no longer stir the highly viscous polymer melt, resulting in all of the copolymers having similar *M*_w_ values (45,200–50,600 g mol^–1^) and monomodal distributions (Figure S5). Polymer yield decreased as the *n*_GA_ increased since more glycolide formed as a side product. From Figure S4, it can be seen that the copolymers darkened in appearance as the *n*_GA_ increased, suggesting the discoloration is due to the thermal degradation of glycolic acid segments in the polymer.

**Table 1:** Molecular Properties of PGBAT, PGBS, and PGBT Copolymers Synthesized on a 30 g Scale^a^

	polycondensation		feed	polymer					
sample	temp/°C	time/h	*n*_GA_/mol %	*n*_GA_/mol %	B:A + T	M_n_/g mol^–1^	M_w_/g mol^–1^	*Đ*	yield/%
PGBAT21	210–230	4	25	21	1:0.99	26,300	50,600	1.92	98
PGBAT44	210–220	5	50	44	1:0.97	18,100	45,200	2.49	96
PGBAT86	210	4.5	90	86	1:1	21,700	46,700	2.15	78
PGBAT89	210	3.5	92	89	1:0.93	24,000	47,300	1.97	75
PGBAT92	210	3.5	94	92	1:0.98	24,500	47,300	1.93	74
PGBS91	210	3.5	90	91	1:0.93^b^	18,700	34,400	1.84	92
PGBT80	210	5	90	80	1:0.90	20,300	40,600	2.00	65

a For PGBAT21–44 feed, B:A + T = 1.25:1; for PGBAT86–92, PGBS91, and PGBT80 feed, B:A + T = 1.05:1.

b B:S value.

PGBS and PGBT with *n*_GA_ feed values of 90% were also synthesized to observe how changing the diacid influenced the copolymers’ properties. Compared to PGBAT86–92, PGBT required a longer polycondensation time to obtain a highly viscous melt and had a lower yield and a lower *n*_GA_ of 80%. Therefore, further reaction optimization is likely required for this copolymer. Conversely, PGBS’s yield was higher, and it showed less discoloration.

**Figure 1:**
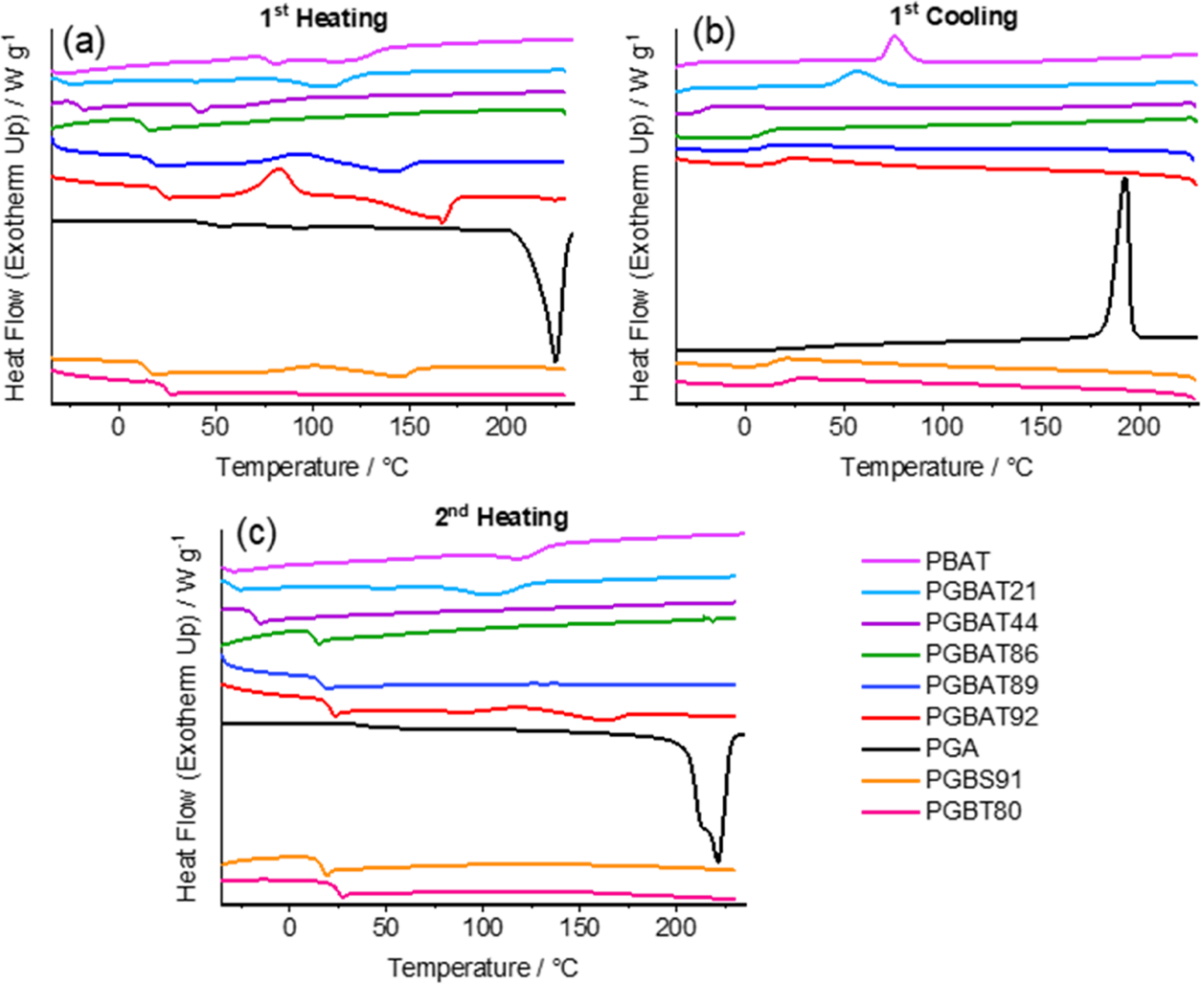
(a) DSC 1st heating curves, (b) DSC 1st cooling curves, and (c) DSC 2nd heating curves of PGBAT, PGBS, PGBT, PGA, and PBAT.

DSC analysis was carried out using samples from the reaction flask, which were stored at room temperature for 5 days. The results (Table 2 and Figure 1) revealed that PGBATs’ *T*_g_ ranged from −19 to 20 °C and increased as more glycolic acid was incorporated. All PGBAT copolymers displayed melting peaks (61.3–166.9 °C) in their first heating cycle, but only PGBAT21 and PGBAT92 showed melting peaks in their second heating cycle. Thus, PGBAT21 and PGBAT92 were semicrystalline, whereas PGBAT44–PGBAT89 were virtually amorphous and had very slow crystallization rates, which is likely due to their lack of structural regularity. This agrees with similar trends observed with increasing *n*_*GA*_ in PGBS and PGBF.^18,19,29^ Unlike PGA, PGBAT92 showed a cold crystallization peak during its second heating cycle but no crystallization during cooling, indicating its slow crystallization rate. Since PGBAT86–92 displayed lower melting temperatures (132.8–166.9 °C) and crystallinity (Δ*H*_m_ = 0.6–32.5 J g^–1^) than PGA (*T*_m_ = 225.1 °C, Δ*H*_m_ = 76.0 J g^–1^), introducing these comonomer units can be used to lower PGA’s *T*_m_ and crystallinity,

**Table 2:** Thermal Properties of PGA-Based Copolymers: PGBAT, PGBS, PGBT, PGA, and PBAT Were Measured by DSC

	first heat		first cool		second heat				
sample	*T*_m_/°C	Δ*H*_m_/J g^–1^	*T*_c_/°C	Δ*H*_c_/J g^–1^	*T*_g_/°C	*T*_cc_/°C	Δ*H*_cc_/J g^–1^	*T*_m_/°C	Δ*H*_m_/J g^–1^
PBAT	117.3	23.0	75.9	17.5	–32.7			118.6	11.0
PGBAT21	111.4	14.9	48.3	16.2	–24.5			102.8	14.2
PGBAT44	61.3	11.6			–19.0				
PGBAT86	132.8	0.6			11.2				
PGBAT89	138.9	9.0			15.9				
PGBAT92	166.9	32.5			20.0	118.3	5.7	163.3	5.37
PGA	225.1	76.0	193.0	76.4	40.1			222.5	77
PGBS91	147	7.8			15.7				
PGBT80					23.7				

Compared with PGBAT92, PGBS91 displayed a lower *T*_g_ (15.7 °C), whereas PGBT80’s *T*_g_ was higher (23.7 °C). This shows that removing the aromatic terephthalic acid units lowers these copolymers’ *T*_g_, while increasing the aromatic content raises it.

**Figure 2:**
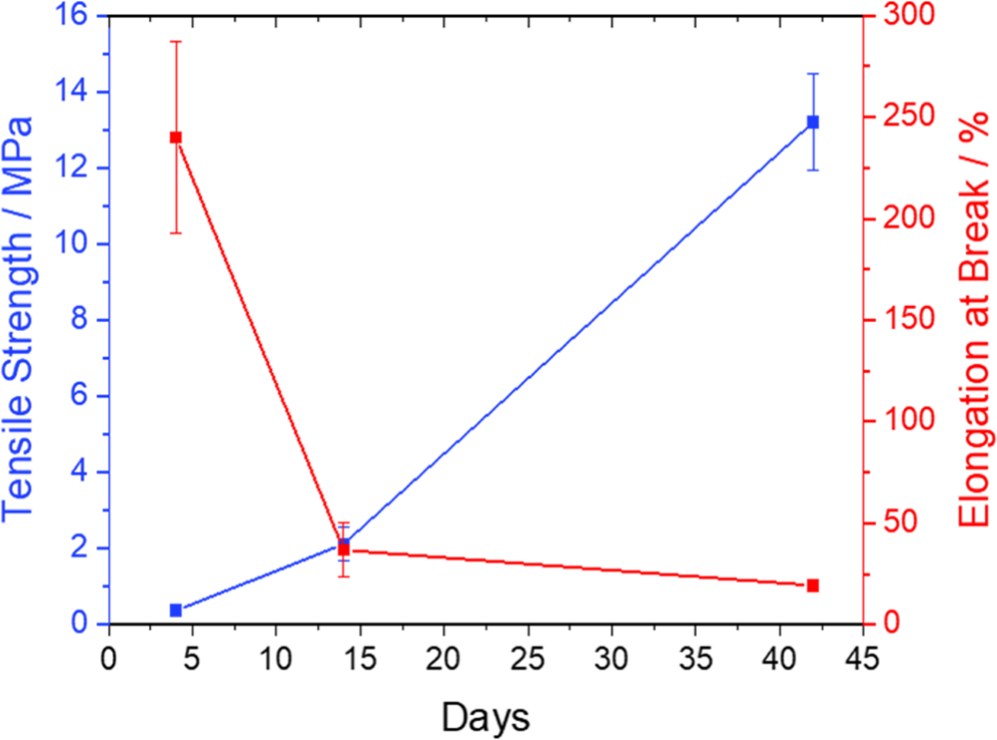
Change in the mechanical properties of PGBAT86 over time stored at 21 °C.

Subsequently, PGBAT films were prepared by hot-pressing; these were then cut into dumbbell-shaped specimens for tensile testing. After hot-pressing, the PGBAT86–89 films appeared soft and sticky but became firmer after storage at room temperature. Therefore, PGBAT86 was stored at 21 °C for 6 weeks, and the changes in its mechanical properties were examined (Figure 2). PGBAT86 changed from a tacky elastomeric polymer to a brittle high-strength material. PGBAT86’s *T*_g_ was just below room temperature, and it showed a weak melting peak in its first DSC heating cycle but not in its second, which suggests the copolymer crystallizes slowly. Poly(butylene carbonate-*co*-furandicarboxylate) copolymers with similar thermal properties have been shown to crystallize during room temperature storage, as do poly(3-hydroxybutyrate) and PGBS.^19,30,31^ Therefore, the observed changes in the mechanical properties of PGBAT86 may be caused by a similar phenomenon.

**Figure 3:**
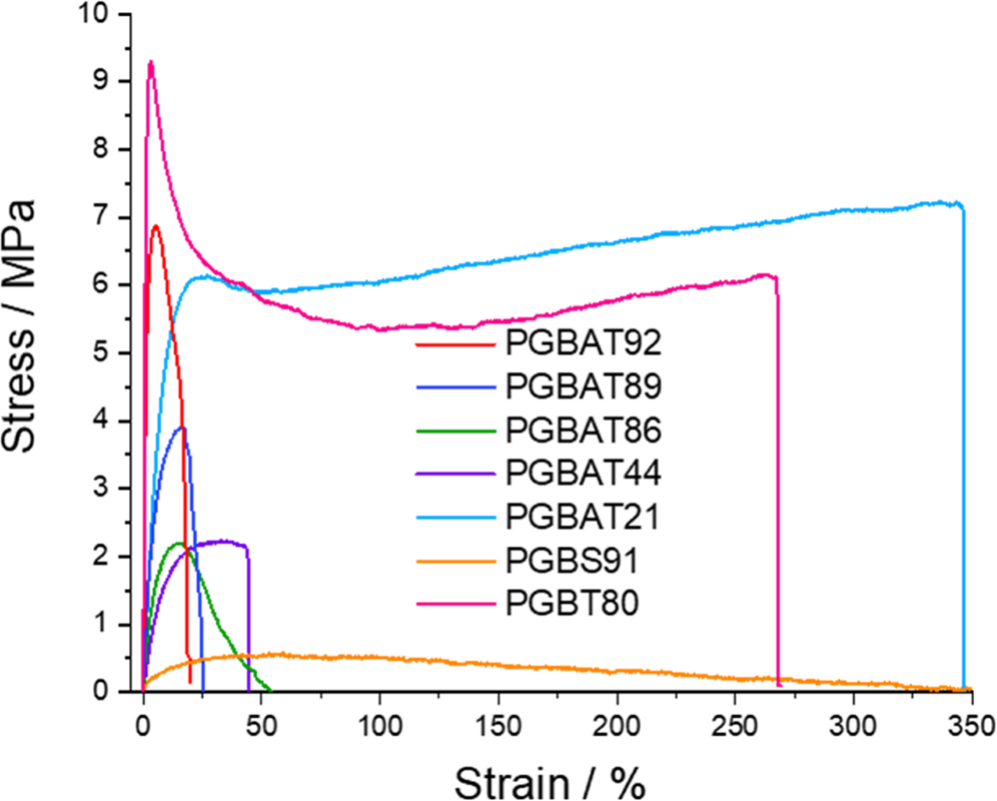
Stress–strain curves of PGBAT, PGBS, and PGBT copolymers.

Tensile testing was then performed on all copolymers (Table 3 and Figure 3), and the copolymers were stored for 2 weeks at 21 °C before undergoing tensile testing to allow for structural relaxation and crystallization. Upon increasing the glycolic acid content in PGBAT from 0 to 21 to 44%, the tensile strength decreased from 24.1 ± 1.9 to 7.23 ± 0.71, then to 2.04 ± 0.26 MPa, and the elongation at break reduced from 774 ± 61 to 329 ± 75 and then to 40.8 ± 8.3%. The introduction of glycolic acid units likely disrupted the structural regularity of the PBAT segments, reducing the crystallinity and leading to poorer mechanical performance. As the *n*_GA_ further increased from 86 to 89 to 92%, the tensile strength increased from 2.12 to 3.79 to 6.14 MPa, and the elongation at break fell from 36.8 to 28.1 to 18.6%. This is the result of the *T*_g_ increasing from 11.2 to 20.0 °C and the polymer chains becoming stiffer and more rigid as more glycolic acid is added. PGA’s tensile strength of 124 MPa was significantly higher, but its elongation at break (3%) was much lower. Therefore, minor amounts of butylene adipate and butylene terephthalate units can be copolymerized with PGA to improve its flexibility at the expense of reductions in strength.

**Table 3:** Mechanical Properties of PGBAT, PGBS, and PGBT Copolymers^a^

sample	Young’s modulus/MPa	tensile strength/MPa	elongation at break/%
PBAT	101 ± 30	24.1 ± 1.9	774 ± 61
PGBAT21	83.9 ± 6.8	7.23 ± 0.71	329 ± 75
PGBAT44	26.5 ± 2.2	2.04 ± 0.26	40.8 ± 8.3
PGBAT86	53.5 ± 9.4	2.12 ± 0.44	36.8 ± 13.2
PGBAT89	74.8 ± 20.8	3.79 ± 1.66	28.1 ± 16.6
PGBAT92	329 ± 56	6.12 ± 0.60	18.6 ± 6.6
PGA	7600 ± 1200	124 ± 21	3.0 ± 5.8
PGBS91	9.74 ± 2.13	0.60 ± 0.08	410 ± 209
PGBT80	674 ± 55	9.10 ± 0.82	285 ± 143

a Samples are compared to commercial PBAT and PGA. Samples were stored for 2 weeks at 21 °C before testing.

Due to the absence of aromatic units, PGBS91 was a softer, tackier material with a tensile strength of 0.57 ± 0.09 MPa and an elongation at break of 496 ± 33%. PGBT80 displayed significantly improved elongation at break (285 ± 140%) and higher tensile strength (8.56 ± 1.40 MPa) compared to PGBAT86–92. This is likely because, unlike the other copolymers, PGBT80s *T*_g_ (23.7 °C) was higher than the room temperature (21 °C), so it was in its glassy state during testing while all of the other copolymers were in their rubbery state.

**Figure 4:**
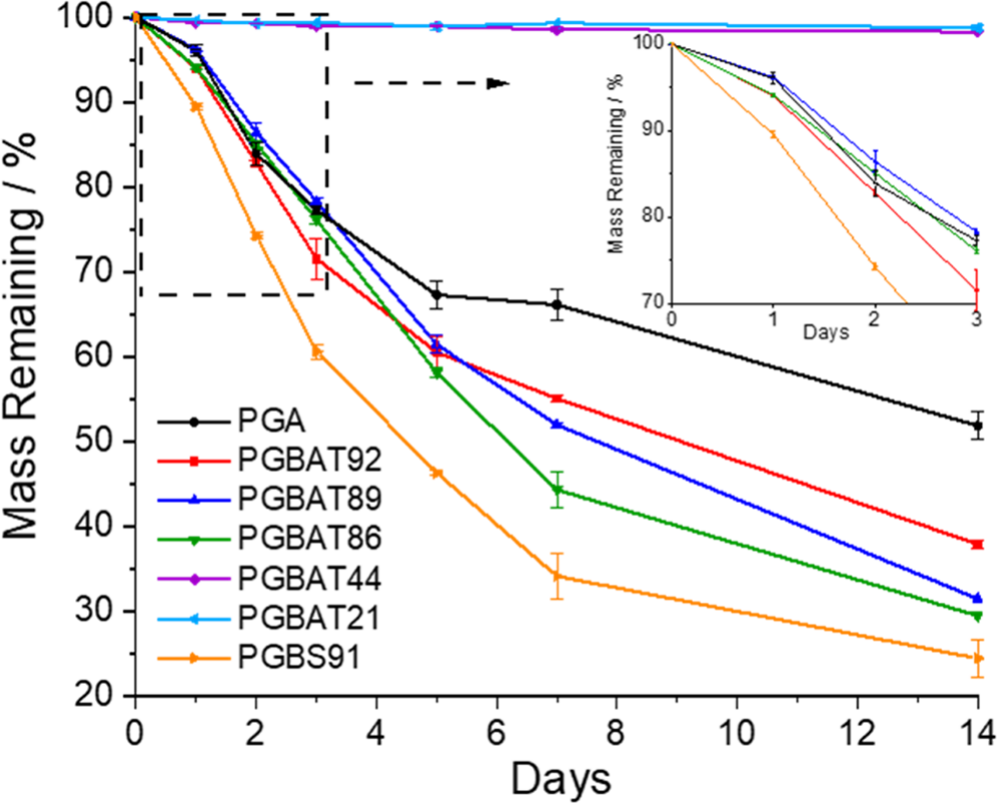
Mass remaining of PGBAT, PGBS, and PGA after 0–14 days of degradation in pH 7 PBS buffer at 60 °C.

In order to compare the hydrolytic stability of these copolymers with that of PGA, hydrolytic degradation tests were performed by measuring the mass loss of samples in PBS buffer at 60 °C. Figure 4 shows that initially (0–3 days), PGBAT86–92 underwent hydrolysis at a similar rate to PGA but then showed much higher mass losses than PGA between 5 and 14 days, suggesting faster hydrolysis. This is likely a result of their lower crystallinity compared to PGA, since amorphous regions are more susceptible to hydrolytic degradation. PGBAT21 and PGBAT44 displayed almost no mass loss over this period, indicating they have much higher hydrolytic stability. This is similar to trends observed in PGBT and PGBF, where the *n*_GA_ was varied from 0–60%; polymers had very slow degradation rates at 0–50% of glycolic acid but showed rapid increases at 60% of glycolic acid.^23,29^

PGBS91 displayed faster hydrolysis than PGBAT86–92, and this was attributed to its lower molecular weight. The mass loss of PGBT80 could not be determined since it melted during testing; this was because it was completely amorphous (no *T*_m_ or *T*_cc_ in DSC first heating cycle) and was heated well above its *T*_g_ (23.7 °C).

### 3.3 Effects of Annealing on the Thermomechanical Properties and Hydrolysis of PGBS and PGBAT

During the previous studies, the increase in molecular weight was limited by the use of magnetic stirring, which cannot effectively stir the reaction mixture above a certain molecular weight due to its high viscosity. To increase molecular weight and explore how annealing affects these polymers’ properties, polymerizations of PGBAT and PGBS with an *n*_GA_ of 90% were repeated using mechanical stirring. Despite its superior mechanical properties, PGBT80 was not studied further due to its amorphous structure and poor heat stability making it unsuitable for annealing.

Under these conditions, the *M*_w_ of PGBS increased significantly, producing a high-molecular-weight polymer with an *M*_w_ of 97,000 g mol^–1^, Table 4. Compared to the previously synthesized PGBAT’s, the PGBAT produced using mechanical stirring did not show a similar *M*_w_ increase; an *M*_w_ of 49,400 g mol^–1^ was achieved, which was significantly lower than PGBS90s. The ^1^H NMR spectra (Figures S6 and S7) revealed these copolymers’ *n*_*GA*_ values were close to the feed value (90%), and SEC analysis showed that they are monomodal (Figure S9). Additionally, as was observed before, PGBS displayed less discoloration and required a shorter reaction time than PGBAT (Figure S8).

**Table 4:** Molecular Properties of PGBS and PGBAT Copolymers Synthesized Using Mechanical Stirring with an *n*_GA_ Feed Value of 90% on a 60 g Scale^a^

		feed	polymer					
sample	time/h	*n*_GA_/mol %	*n*_GA_/mol %	B:A + T	*M*_n_/g mol^–1^	*M*_w_/g mol^–1^	*Đ*	yield/%
PGBS90	4	90	90	1:0.97^b^	54,800	97,000	1.77	97
PGBAT88	8	90	88	1:1.04	19,500	49,400	2.53	95

a Polycondensation temperature was 210 °C. Both copolymers were synthesized using a B:A + T = 1.05:1.

b B:S value.

**Figure 5:**
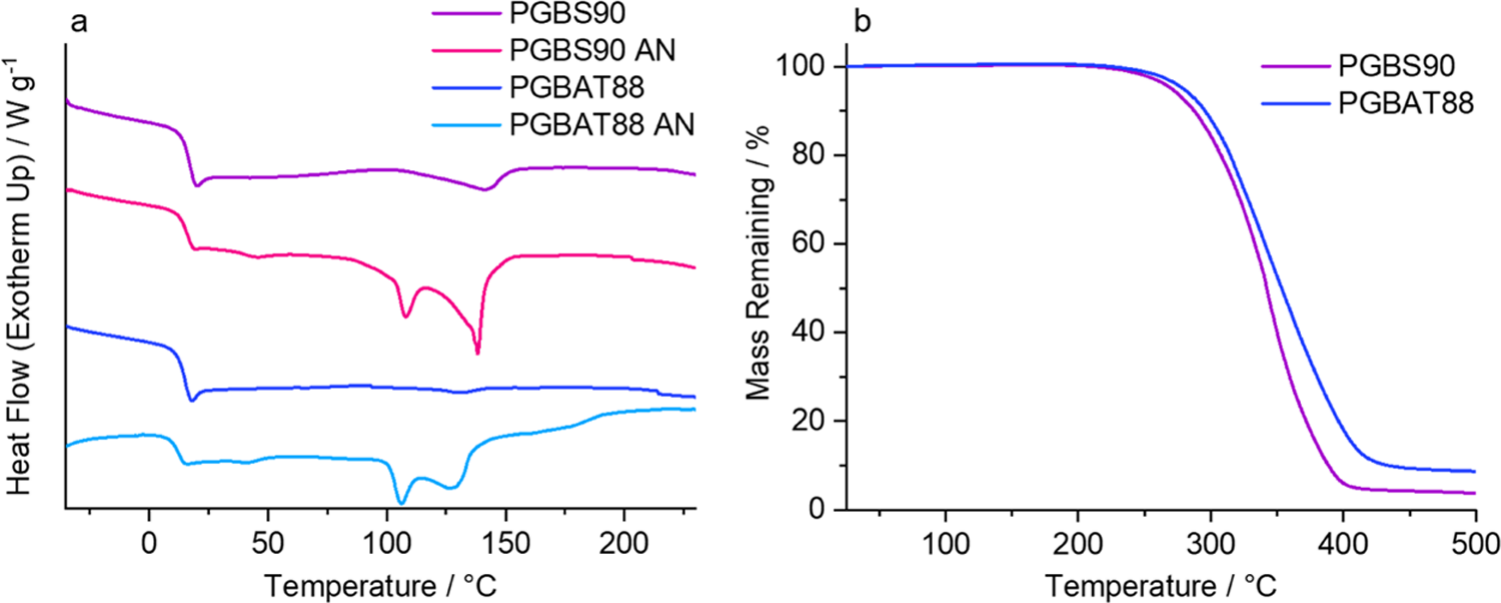
(a) DSC 1st heating curves of amorphous and annealed PGBS90 and PGBAT88 copolymers. (b) TGA Thermograms of PGBS90 and PGBAT88.

In the DSC (Table 5 and Figure 5), these copolymers displayed very weak cold crystallization and melting peaks in their first heating cycles, suggesting they possessed some degree of crystallinity (<2%) and could crystallize under certain conditions. Therefore, film samples were annealed for 1 h at 100 °C to increase their crystallinity. Annealing significantly increased the Δ*H*_m_ values, resulting in a crystallinity increase from 1.8 to 15.5% for PGBS90 and from 0.7 to 8.0% for PGBAT88. After annealing, these copolymers’ appearances changed (Figure S8), turning from transparent to opaque, which further indicates a transition from an amorphous state to a semicrystalline one. These copolymers had melting temperatures of 127.6–141.0 °C, well below that of PGA (225.1 °C), so they can be processed at significantly lower temperatures. TGA showed they have *T*_d 5%_ values of 271–280 °C, so they have a wide processing window (*T*_m_ – *T*_d_).

**Table 5:** Thermal Properties of PGBS90 and PGBAT88 before and after Annealing

	first heat					second heat	TGA
sample	*T*_cc_/°C	Δ*H*_cc_/J g^–1^	*T*_m_/°C	Δ**H**_m_/J g^-1^	*X*_c_^a^/%	*T*_g_/°C	*T*_d 5%_/°C
PGBS90	98.6	2.2	141.0	5.5	1.8	16.8	271
PGBS90 AN			138.0	28.5	15.5	16.6	
PGBAT88			131.8	1.4	0.7	15.1	280
PGBAT88	59.6	1.7	127.6	16.3	8.0	15.1	

a The percentage of crystallinity was calculated using *X*_c_ = (Δ*H*_m_ – Δ*H*_cc_)/Δ*H*_m_°_PGA_ × 100%, where Δ*H*_m_°_PGA_ = 183.2 J g^–1^.^32^

**Figure 6:**
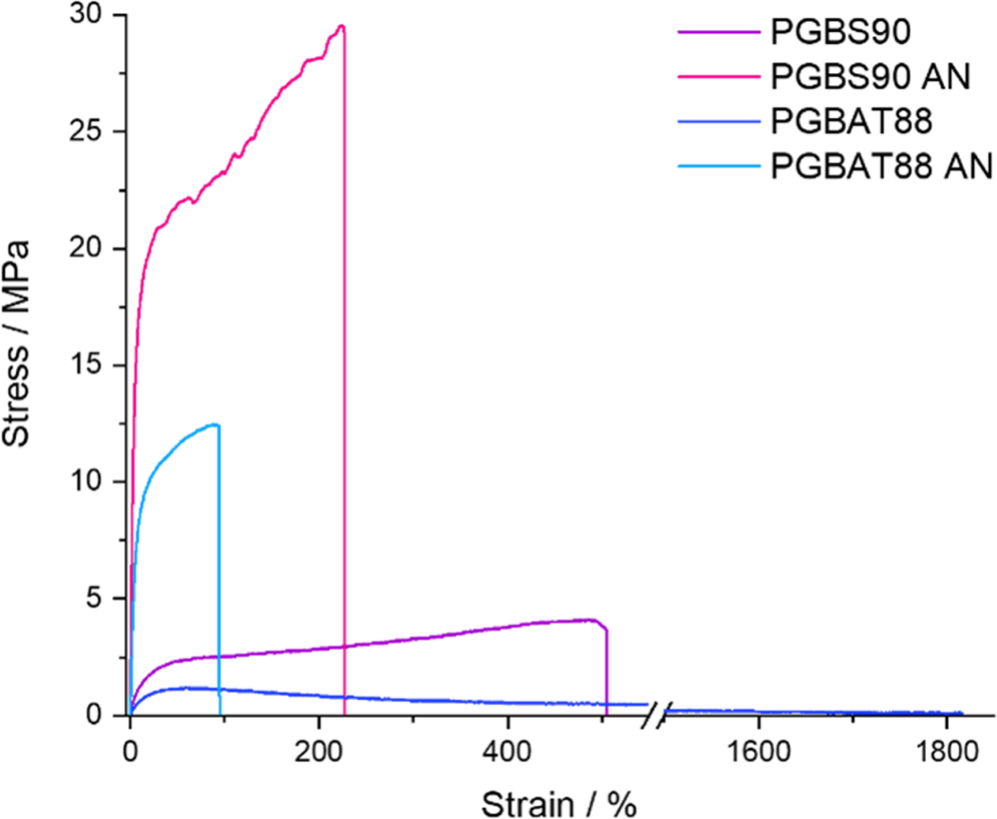
Stress–strain curves of amorphous and annealed PGBS90 and PGBAT88 copolymers.

The mechanical properties of PGBS90 and PGBAT88 changed significantly after annealing (Table 6 and Figure 6). Amorphous PGBS90 and PGBAT88 were highly flexible elastomeric materials, which became tougher and stiffer following annealing. Annealed PGBS88 showed improved properties; its modulus and tensile strength increased from 40.5 to 588 MPa and from 4.19 to 30.0 MPa, but its elongation at break fell from 537 to 171%. Annealed PGBAT88 displayed similar increases, but its modulus (189 ± 24 MPa), tensile strength (11.8 ± 0.5 MPa), and elongation at break (85.9 ± 21.6%) were lower, which was likely due to its lower molecular weight. These annealed copolymers showed excellent elongation at break in comparison to PGA (elongation 3.0 ± 5.8%) while still maintaining high tensile strength and modulus. Therefore, annealing can be used to produce PGA copolymers containing ∼90% *n*_GA_ with high strength and increased flexibility over PGA.

**Table 6:** Mechanical Properties of PGBS90 and PGBAT88 before and after Annealing^a^

sample	Young’s modulus/MPa	tensile strength/MPa	elongation at break/%
PGBS90	40.5 ± 17.9	4.19 ± 1.21	537 ± 71
PGBS90 AN	588 ± 69	30.0 ± 2.5	171 ± 41
PGBAT88	10.9 ± 1.6	1.10 ± 0.01	1820^b^
PGBAT88 AN	189 ± 24	11.8 ± 0.5	85.9 ± 21.6

a Samples were stored at 21 °C for 2 days before testing.

b All samples reached maximum extension without breaking.

From the ^1^H NMR spectrum (Figure S7), it was calculated that PGBS90 contained approximately 75 wt % of PGA. Compared to compatibilized PGA blends containing similar PGA quantities, PGBS90 displayed superior elongation at break. 70/30 PGA/PBAT blends were reported with an elongation at break of 45% and a tensile strength of 46 MPa.^33^ 70/30 PGA/PCL blends showed an elongation at break of 40.3% and a tensile strength of 49.6 MPa.^34^

**Figure 7:**
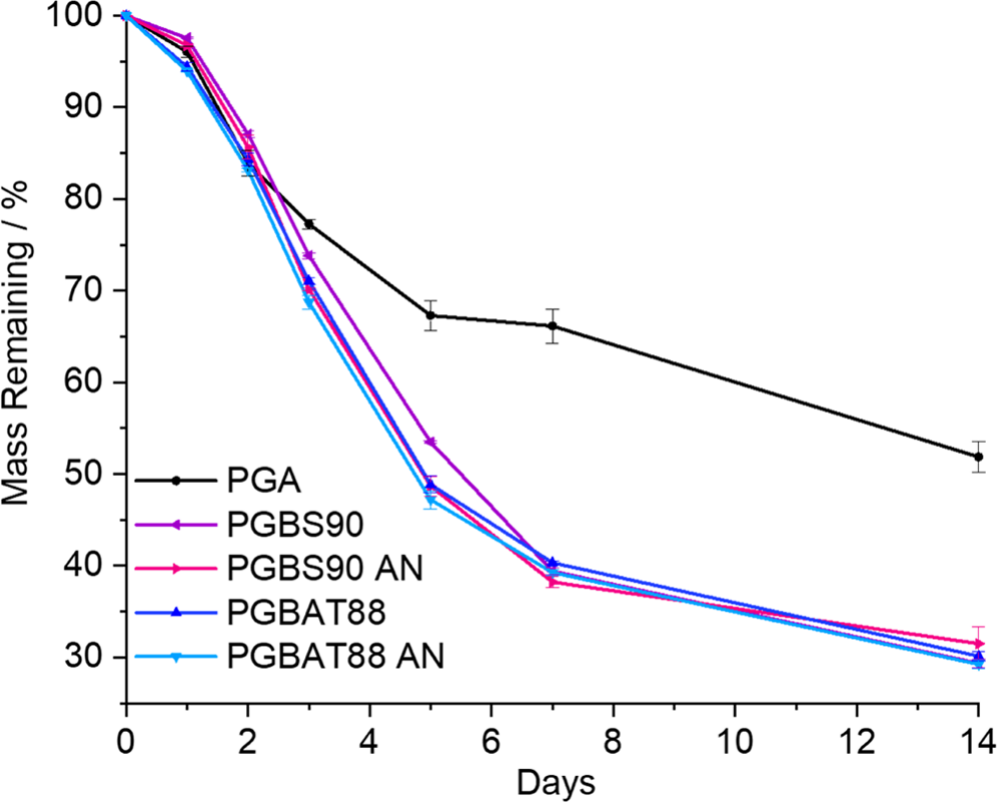
Mass remaining of amorphous and annealed PGBS90 and PGBAT88 copolymers after 0–14 days of degradation in pH 7 PBS buffer at 60 °C.

It is known that annealing PGA can produce more ordered crystalline structures, which prevent the intrusion of water molecules, improving hydrolytic stability.^35−37^ Therefore, it was investigated whether annealing influenced the rates of hydrolysis. There were no significant differences in the hydrolysis rate between the annealed and amorphous samples of PGBS90 and PGBAT88, Figure 7. As was observed in the previous tests, during the initial stages, they showed mass losses similar to PGA, but after 5 days, they showed significantly increased mass loss to PGA, indicating faster hydrolysis. Both PGBS90 and PGBAT88 displayed nearly identical rates of mass loss under these test conditions. During degradation, the amorphous samples turned from transparent to opaque, indicating an increase in crystallinity, suggesting that annealing occurred.

## 4 Conclusions

PGBAT, PGBS, and PGBT copolymers containing glycolic acid as their major component were successfully synthesized by melt polycondensation. The synthesis of PGBAT from the direct esterification and polycondensation of monomers produced higher yields and molecular weights than those from the polycondensation of oligomers. Additionally, the ratio of diol to diacid was found to significantly affect the molecular weight. In PGBAT21–92, the mechanical properties were highly dependent on the comonomer ratio. As the *n*_GA_ value increased, elongation at break fell, and copolymers became more amorphous. At very high *n*_GA_ values (86–92%), PGBAT displayed improved elongation at break over PGA (18.6–36.8% vs 3%) but reduced tensile strength (2.12–6.12 MPa vs 124 MPa).

The mechanical properties of PGBAT88 (*M*_w_ 49,400 g mol^–1^) and PGBS90 (*M*_w_ 97,000 g mol^–1^) were largely dependent upon their crystallinity. In their amorphous states, these copolymers were weak, elastomeric materials (tensile strength 1.10–4.19 MPa, elongation at break 537–1820%); however, after annealing at 100 °C, they became semicrystalline and displayed increased strength (11.8–30.0 MPa) and decreased elongation (85.9–171%). Hydrolysis tests performed at 60 °C in PBS buffer showed that these copolymers degrade at similar rates to PGA during the early stages of degradation but then proceed to degrade much more rapidly than PGA. Overall, this work shows that high *n*_GA_ PGBS and PGBAT copolymers can be synthesized and annealed to yield materials with high strength and improved flexibility over PGA.

## Supporting Information Available

Appearance of PGBAT copolymers synthesized using different polycondensation catalysts (Figure S1); appearance of PGBAT copolymers synthesized using different esterification catalysts (Figure S2); appearance of PGBAT copolymers synthesized using different antioxidants (Figure S3); appearance of PGBAT copolymers (Figure S4); SEC traces of PGBAT copolymers (Figure S5); ^1^H NMR (400 MHz) spectrum of PGBAT88 copolymer in DMSO-*d*_6_ (Figure S6); ^1^H NMR (400 MHz) spectrum of PGBS90 copolymer in DMSO-*d*_6_ (Figure S7); appearance of amorphous and annealed films of PGBS90 and PGBAT88 (Figure S8); and SEC traces of PGBS90 and PGBAT88 in DMF as eluent (Figure S9). Catalyst screen for synthesis of PGBAT50 (Table S1); the effect of varying the catalyst used for the esterification of glycolic acid upon the final polycondensation reaction (Table S2); screening of common antioxidants for their ability to reduce discoloration during PGBAT50 synthesis (Table S3); molecular properties and synthesis conditions of PGBAT copolymers containing high nGA values (Table S4); thermomechanical properties of PGBAT copolymers containing high nGA values (Table S5); PGBAT copolymers synthesized via the direct esterification and polycondensation of monomers (Table S6); and the effects of varying the ratio of 1,4-butanediol to adipic acid and dimethyl terephthalate (B:A + T) on the molecular weight of PGBAT90 (Table S7) (PDF)

## References

[ref1] Yamane K.; Sato H.; Ichikawa Y.; Sunagawa K.; Shigaki Y. Development of an industrial production technology for high-molecular-weight polyglycolic acid. Polym. J. 2014, 46 (11), 769–775. 10.1038/pj.2014.69.

[ref2] Kureha K. Polyglycolic acid (PGA) Resin Product Brochure. https://www.kureha.co.jp/en/business/material/pdf/Kuredux_en.pdf.

[ref3] de Beukelaer H.; Hilhorst M.; Workala Y.; Maaskant E.; Post W. Overview of the mechanical, thermal and barrier properties of biobased and/or biodegradable thermoplastic materials. Polym. Test. 2022, 116, 107803 10.1016/j.polymertesting.2022.107803.

[ref4] Samantaray P. K.; Little A.; Haddleton D.; McNally T.; Tan B.; Sun Z.; Huang W.; Ji Y.; Wan C. Poly (glycolic acid)(PGA): a versatile building block expanding high performance and sustainable Bioplastic applications. Green Chem. 2020, 22, 4055–4081. 10.1039/D0GC01394C.

[ref5] Jem K. J.; Tan B. The Development and Challenges of Poly (lactic acid) and Poly (glycolic acid). Adv. Ind. Eng. Polym. Res. 2020, 3, 60–70. 10.1016/j.aiepr.2020.01.002.

[ref6] Ellingford C.; Samantaray P. K.; Farris S.; McNally T.; Tan B.; Sun Z.; Huang W.; Ji Y.; Wan C. Reactive extrusion of biodegradable PGA/PBAT blends to enhance flexibility and gas barrier properties. J. Appl. Polym. Sci. 2022, 139 (6), 51617.

[ref7] Samantaray P. K.; Ellingford C.; Farris S.; O’Sullivan D.; Tan B.; Sun Z.; McNally T.; Wan C. Electron beam-mediated cross-linking of blown film-extruded biodegradable PGA/PBAT blends toward high toughness and low oxygen permeation. ACS Sustainable Chem. Eng. 2022, 10 (3), 1267–1276. 10.1021/acssuschemeng.1c07376.

[ref8] Yang F.; Zhang C.; Ma Z.; Weng Y. In situ formation of microfibrillar PBAT in PGA films: an effective way to robust barrier and mechanical properties for fully biodegradable packaging films. ACS Omega 2022, 7 (24), 21280–21290. 10.1021/acsomega.2c02484. 35935288 PMC9348010

[ref9] Dobrzynski P. Synthesis of biodegradable copolymers with low-toxicity zirconium compounds. III. Synthesis and chain-microstructure analysis of terpolymer obtained from L-lactide, glycolide, and ϵ-caprolactone initiated by zirconium (IV) acetylacetonate. J. Polym. Sci., Part A: Polym. Chem. 2002, 40 (18), 3129–3143. 10.1002/pola.10401.

[ref10] Zurita R.; Puiggalí J.; Franco L.; Rodríguez-Galán A. Copolymerization of glycolide and trimethylene carbonate. J. Polym. Sci., Part A: Polym. Chem. 2006, 44 (2), 993–1013. 10.1002/pola.21199.

[ref11] Kuehster L.; Jhon Y. K.; Wang Y.; Smith W. C.; Xu X.; Qin B.; Zhang F.; Lynd N. A. Stochastic and Deterministic Analysis of Reactivity Ratios in the Partially Reversible Copolymerization of Lactide and Glycolide. Macromolecules 2022, 55 (16), 7171–7180. 10.1021/acs.macromol.2c00757.

[ref12] Olewnik E.; Czerwiński W. Synthesis, structural study and hydrolytic degradation of copolymer based on glycolic acid and bis-2-hydroxyethyl terephthalate. Polym. Degrad. Stab. 2009, 94 (2), 221–226. 10.1016/j.polymdegradstab.2008.10.026.

[ref13] Zhou J.; Zhu Q.; Pan W.; Xiang H.; Hu Z.; Zhu M. Thermal Stability of Bio-Based Aliphatic-Semiaromatic Copolyester for Melt-Spun Fibers with Excellent Mechanical Properties. Macromol. Rapid Commun. 2021, 42 (3), 2000498 10.1002/marc.202000498. 33336853

[ref14] Nakayama Y.; Yagumo W.; Tanaka R.; Shiono T.; Inumaru K.; Tsutsumi C.; Kawasaki N.; Yamano N.; Nakayama A. Synthesis, properties and biodegradation of periodic copolyesters composed of hydroxy acids, ethylene glycol, and terephthalic acid. Polym. Degrad. Stab. 2020, 174, 109095 10.1016/j.polymdegradstab.2020.109095.

[ref15] Bacskai R. Flexible Glycolic Acid Terpolymers. US4139525A, 1977.

[ref16] Ruan L.; L W.; Tang Y.; He X.; Yang W.; Mao B. Method for synthesizing pbat-pla copolyester by means of copolymerization. EP3453731A1, 2017.

[ref17] Wang G.; J J.; Huang D. Hydrolyzable copolyester, preparation method therefor, and application thereof. WO2020156346A1, 2021.

[ref18] Ding Y.; Huang D.; Ai T.; Zhang C.; Chen Y.; Luo C.; Zhou Y.; Yao B.; Dong L.; Du X. Bio-based poly (butylene furandicarboxylate-co-glycolate) copolyesters: synthesis, properties, and hydrolysis in different aquatic environments for water degradation application. ACS Sustainable Chem. Eng. 2021, 9 (3), 1254–1263. 10.1021/acssuschemeng.0c07351.

[ref19] Hu H.; Li J.; Tian Y.; Chen C.; Li F.; Ying W. B.; Zhang R.; Zhu J. Experimental and theoretical study on glycolic acid provided fast bio/seawater-degradable poly (butylene succinate-co-glycolate). ACS Sustainable Chem. Eng. 2021, 9 (10), 3850–3859. 10.1021/acssuschemeng.0c08939.

[ref20] Liu T.-Y.; Xu P.-Y.; Huang D.; Lu B.; Zhen Z.-C.; Zheng W.-Z.; Dong Y.-C.; Li X.; Wang G.-X.; Ji J.-H. Enhanced degradation of poly (ethylene terephthalate) by the addition of lactic acid/glycolic acid: composting degradation, seawater degradation behavior and comparison of degradation mechanism. J. Hazard. Mater. 2023, 446, 130670 10.1016/j.jhazmat.2022.130670. 36580787

[ref21] Wang Y.; Yang H.; Li B.; Liu S.; He M.; Chen Q.; Li J. Poly (Butylene Adipate/Terephthalate-Co-Glycolate) Copolyester Synthesis Based on Methyl Glycolate with Improved Barrier Properties: From Synthesis to Structure-Property. Int. J. Mol. Sci. 2022, 23 (19), 11074 10.3390/ijms231911074. 36232379 PMC9570190

[ref22] Ding Y.; Wang J.; Luo C.; Yao B.; Dong L.; Du X.; Ji J. Modification of poly (butylene succinate) with biodegradable glycolic acid: Significantly improved hydrolysis rate retaining high toughness property. J. Appl. Polym. Sci. 2022, 139 (19), 52106 10.1002/app.52106.

[ref23] Wang Y.; Liu J.; Li C.; Xiao Y.; Wu S.; Zhang B. Synthesis and characterization of poly (butylene terephthalate-co-glycolic acid) biodegradable copolyesters. Eur. Polym. J. 2022, 180, 111613 10.1016/j.eurpolymj.2022.111613.

[ref24] Hu H.; Zhang R.; Ying W. B.; Kong Z.; Wang K.; Wang J.; Zhu J. Biodegradable elastomer from 2, 5-furandicarboxylic acid and ε-caprolactone: effect of crystallization on elasticity. ACS Sustainable Chem. Eng. 2019, 7 (21), 17778–17788. 10.1021/acssuschemeng.9b04210.

[ref25] Xu P.-Y.; Liu T.-Y.; Huang D.; Zhen Z.-C.; Lu B.; Li X.; Zheng W.-Z.; Wang G.-X.; Ji J.-H. Degradation performances of CL-modified PBSCL copolyesters in different environments. Eur. Polym. J. 2022, 174, 111322 10.1016/j.eurpolymj.2022.111322.

[ref26] Liu T.-Y.; Huang D.; Xu P.-Y.; Lu B.; Wang G.-X.; Zhen Z.-C.; Ji J. Biobased Seawater-Degradable Poly (butylene succinate-l-lactide) Copolyesters: Exploration of Degradation Performance and Degradation Mechanism in Natural Seawater. ACS Sustainable Chem. Eng. 2022, 10 (10), 3191–3202. 10.1021/acssuschemeng.1c07176.

[ref27] Hu H.; Zhang R.; Shi L.; Ying W. B.; Wang J.; Zhu J. Modification of poly (butylene 2, 5-furandicarboxylate) with lactic acid for biodegradable copolyesters with good mechanical and barrier properties. Ind. Eng. Chem. Res. 2018, 57 (32), 11020–11030. 10.1021/acs.iecr.8b02169.

[ref28] Murcia Valderrama M. A.; van Putten R.-J.; Gruter G.-J. M. PLGA Barrier Materials from CO2. The influence of Lactide Co-monomer on Glycolic Acid Polyesters. ACS Appl. Polym. Mater. 2020, 2 (7), 2706–2718. 10.1021/acsapm.0c00315. 32954354 PMC7493221

[ref29] Hu H.; Zhang R.; Wang J.; Ying W. B.; Shi L.; Yao C.; Kong Z.; Wang K.; Zhu J. A mild method to prepare high molecular weight poly (butylene furandicarboxylate-co-glycolate) copolyesters: effects of the glycolate content on thermal, mechanical, and barrier properties and biodegradability. Green Chem. 2019, 21 (11), 3013–3022. 10.1039/C9GC00668K.

[ref30] Hu H.; Zhang R.; Wang J.; Ying W. B.; Zhu J. Synthesis and structure–property relationship of biobased biodegradable poly (butylene carbonate-co-furandicarboxylate). ACS Sustainable Chem. Eng. 2018, 6 (6), 7488–7498. 10.1021/acssuschemeng.8b00174.

[ref31] Jenkins M. J.; Robbins K. E.; Kelly C. A. Secondary crystallisation and degradation in P (3HB-co-3HV): An assessment of long-term stability. Polym. J. 2018, 50 (5), 365–373. 10.1038/s41428-017-0012-8.

[ref32] Nakafuku C.; Yoshimura H. Melting parameters of poly (glycolic acid). Polymer 2004, 45 (11), 3583–3585. 10.1016/j.polymer.2004.03.041.

[ref33] Niu D.; Xu P.; Sun Z.; Yang W.; Dong W.; Ji Y.; Liu T.; Du M.; Lemstra P. J.; Ma P. Superior toughened bio-compostable Poly (glycolic acid)-based blends with enhanced melt strength via selective interfacial localization of in-situ grafted copolymers. Polymer 2021, 235, 124269 10.1016/j.polymer.2021.124269.

[ref34] Xu P.; Tan S.; Niu D.; Yang W.; Ma P. Highly toughened sustainable green polyglycolic acid/polycaprolactone blends with balanced strength: morphology evolution, interfacial compatibilization, and mechanism. ACS Appl. Polym. Mater. 2022, 4 (8), 5772–5780. 10.1021/acsapm.2c00715.

[ref35] Browning A.; Chu C. The effect of annealing treatments on the tensile properties and hydrolytic degradative properties of polyglycolic acid sutures. J. Biomed. Mater. Res. 1986, 20 (5), 613–632. 10.1002/jbm.820200507. 3011808

[ref36] Miao Y.; Cui H.; Dong Z.; Ouyang Y.; Li Y.; Huang Q.; Wang Z. Structural Evolution of Polyglycolide and Poly (glycolide-co-lactide) Fibers during In Vitro Degradation with Different Heat-Setting Temperatures. ACS Omega 2021, 6 (43), 29254–29266. 10.1021/acsomega.1c04974. 34746613 PMC8567347

[ref37] Niu D.; Li J.; Xu P.; Liu T.; Yang W.; Wang Z.; Ma P. High-performance and durable fibrous poly (glycolic acid)/poly (butylene adipate-co-terephthalate) blends by reactive compatibilization and solid-state drawing. Polym. Degrad. Stab. 2023, 210, 110293 10.1016/j.polymdegradstab.2023.110293.

