# Supplementary material for: Synthesis and Characterization of High Glycolic Acid Content Poly(glycolic acid-co-butylene adipate-co-butylene terephthalate) and Poly(glycolic acid-co-butylene succinate) Copolymers with Improved Elasticity
Source: ACS Omega. 2023 Oct 3;8(41):38658–67. doi: 10.1021/acsomega.3c05932 (PMC10586444; doi:10.1021/acsomega.3c05932)
Supplement: Supplementary file 1 — ao3c05932_si_001.pdf [file ao3c05932_si_001.pdf]

Supplementary Information

Synthesis and Characterization of High Glycolic Acid Content Poly(glycolic acid-*co*-butylene adipate-*co*-butylene terephthalate) and Poly(glycolic acid-*co*-butylene succinate) Copolymers with Improved Elasticity

Alastair Little[1], Shiyue Ma[1], David M. Haddleton[2], Bowen Tan[3], Zhaoyang Sun[3], Chaoying Wan[1]*

[1]*International Institute for Nanocomposites Manufacturing (IINM), WMG, University of Warwick, CV4 7AL, UK*

[2]*Department of Chemistry, University of Warwick, CV4 7AL, UK*

[3] *PJIM Polymer Scientific Co., Ltd., Shanghai, 201102, China*

Table S1. Catalyst screen for the synthesis of PGBAT50. All copolymers were synthesised via the polycondensation of oligomers at 190 °C for 5 hours at ≤ 0.01 mbar using 0.25 mol % of catalyst on a 2 g scale.

**Catalyst**	$\boldsymbol{n_{GA}}$ / **mol %**	$\mathbf{M_n}$ / **g mol^{-1}**	$\mathbf{M_w}$ / **g mol^{-1}**	**Đ**	**Colour**
Sb_2O_3	50	17,200	34,900	2.03	Brown
$Ti(OBu)_4$	52	10,600	22,200	2.10	Brown
$Sn(Oct)_2$	44	9,060	18,510	2.04	Light brown
None	40	8,560	17,000	1.98	Light brown
BiSS	41	7,640	17,700	2.32	Black
$Zr(acac)_4$	44	7,450	14,900	2.00	Light brown
$Zn(OAc)_2.2H_2O$	39	5,510	10,800	1.97	Light brown

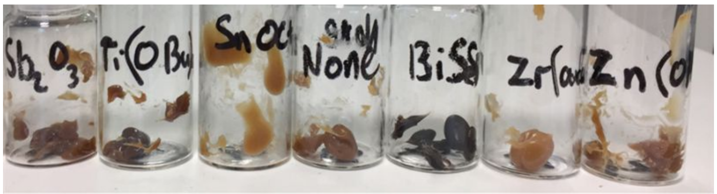

Figure S1. Appearance of PGBAT copolymers synthesised using different polycondensation catalysts.

Table S2. The effect of varying the catalyst used for the esterification of glycolic acid upon the final polycondensation reaction. All copolymers were synthesised at 190 °C for 5 hours at ≤ 0.01 mbar using 0.15 wt % of Sb_2O_3 as the polycondensation catalyst on a 2 g scale.

**OGA**			**PGBAT**		
**Catalyst**	$\boldsymbol{n_{GA}}$ / **mol %**	$\mathbf{M_n}$ / **g mol^{-1}**	$\mathbf{M_w}$ / **g mol^{-1}**	**Đ**	**Colour**
$Zn(acac)_2$	50	17,200	34,900	2.03	Brown
MSA	55	22,300	61,600	2.76	Dark Brown
$Sn(Oct)_2$	48	24,100	105,000	4.38	Dark Brown

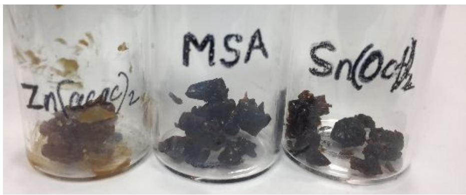

Figure S2. Appearance of PGBAT copolymers synthesised using different esterification catalysts.

Table S3. Screening of common antioxidants for their ability to reduce discolouration during PGBAT50 synthesis. All copolymers were synthesised at 190 °C for 5 hours at ≤ 0.01 mbar using Sb_2O_3 and 0.2 wt % antioxidant on a 2 g scale.

Antioxidant	n_{GA} / mol %	M_n / g mol^{-1}	M_w / g mol^{-1}	*Đ*	Colour
None	48	24,100	105,000	4.38	Dark Brown
Irgafos 168	40	24,400	70,200	2.88	Dark Brown
Irgafos 126	42	24,400	51,000	2.09	Light Brown
H_3PO_4	37	26,900	54,300	2.02	Light Brown

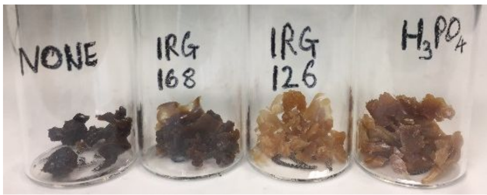

Figure S3. Appearance of PGBAT copolymers synthesised using different antioxidants.

Table S4. Molecular properties and synthesis conditions of PGBAT copolymers containing high n_{GA} values.

Feed n_{GA} / mol %	Polymer n_{GA} / mol %	Polycondensation Temp / °C	Time / h	M_n / g mol^{-1}	M_w / g mol^{-1}	*Đ*	Yield / %
50	45	190	5	32,400	143,000	4.41	92
70	65	190	5	24,800	107,000	4.32	64
90	81	200–210	4	23,200	94,600	4.09	27
80	85	190	6	11,000	21,100	1.91	60
90	95	190–200	2	3,760	6,430	1.71	99

Once the feed n_{GA} was increased to 90 mol %, the reaction temperature had to be raised to 210 °C, since the reaction mixture solidified after 2 hours due to its high melting point (201.3 °C), this further encouraged glycolide formation.

Table S5. Thermomechanical properties of PGBAT copolymers containing high n_{GA} values.

n_{GA} / mol %	T_g / °C	T_c / °C	T_m / °C	Youngs Modulus / MPa	Tensile Strength / MPa	Elongation at break / %
45	-15.7	-	-	6.47 ± 1.73	0.335 ± 0.013	25.2 ± 9.6
65	-7.48	-	-	0.97 ± 0.18	0.0470 ± 0.001	638 ± 73
81	2.24	-	-	0.012 ± 0.002	0.093 ± 0.022	582 ± 260
85	4.53	-	-	369 ± 51	7.29 ± 0.69	21.3 ± 9.4
95	19.3	84.6	201.3	-*	-*	- *

*Could not be tested due to being too brittle

PGBAT45 was soft and tacky with a low tensile strength (0.34 ± 0.01 MPa) and elongation at break (25.2 ± 9.6%). Increasing the n_{GA} to 65% formed an even tackier material with a raised T_g of -7.48 °C, but lower tensile strength (0.047 MPa) and higher elongation at break (638%). PGBAT81 displayed similar mechanical properties and a higher T_g of 2.24 °C. At PGBAT85, the copolymer became firmer, displaying a tensile strength of 7.29 ± 0.69 MPa and elongation at break of 21.3 ± 9.40%. PGBAT45–85 were all amorphous (displayed no T_m during their second DSC heating cycle), whereas PGBAT95 was semi-crystalline, but too brittle to be cut into tensile specimens.

Table S6. PGBAT copolymers synthesised via the direct esterification and polycondensation of monomers. All copolymers were synthesised using $Ti(OBu)_4$ as catalyst at 190–210 °C unless stated otherwise.

Feed		Polymer						
n_{GA} / mol %	B:A+T	n_{GA} / mol %	B:A+T	M_n / g mol^{-1}	M_w / g mol^{-1}	Đ	Yield / %	Appearance
25	1.25:1	20	1:0.98	24,000	44,600	1.85	98	Beige tough solid
50	1.25:1	44	1:0.97	18,100	45,200	2.49	96	Brown tacky solid
80	1.25:1	80	1:0.66	6,570	10,800	1.64	94	Black, sticky liquid
90*	1.25:1	86	1:0.83	16,100	35,800	2.22	84	Black, tacky solid

* Synthesised using $Ti(OBu)_4$, $Sn(Oct)_2$ and Sb_2O_3

Table S7. The effects of varying the ratio of 1,4-butanediol to adipic acid and dimethyl terephthalate (B:A+T) on the molecular weight of PGBAT90. Both copolymers were synthesised using a polycondensation temperature of 210 °C for 4.5 hours.

Feed		Polymer						
n_{GA} / mol %	B:A+T	n_{GA} / mol %	B:A+T	M_n / g mol^{-1}	M_w / g mol^{-1}	Đ	Yield / %	T_g / °C
90	1.25:1	86	1:0.83	16,100	35,800	2.22	84	7.47
90	1.05:1	86	1:1.00	21,700	46,700	2.15	78	11.2

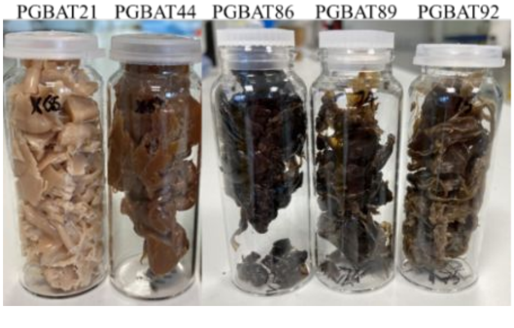

Figure S4. Appearance of PGBAT copolymers.

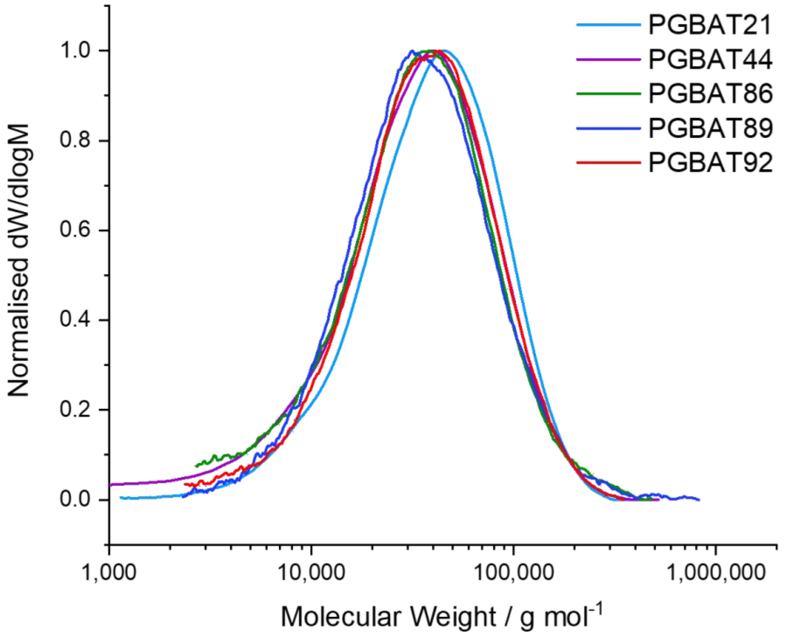

Figure S5. SEC traces of PGBAT copolymers. PGBAT21–44 were run in $CHCl_3$ as eluent, PGBAT86–92 were run in DMF as eluent.

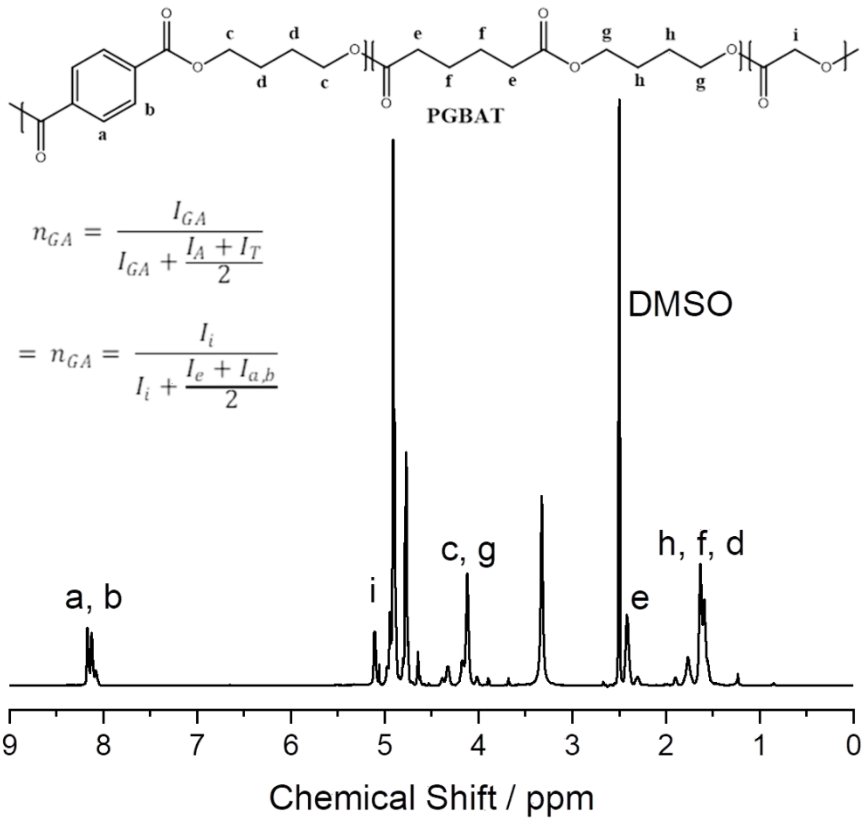

Figure S6. ^{1}H NMR (400 MHz) spectrum of PGBAT88 copolymer in DMSO-d_6.

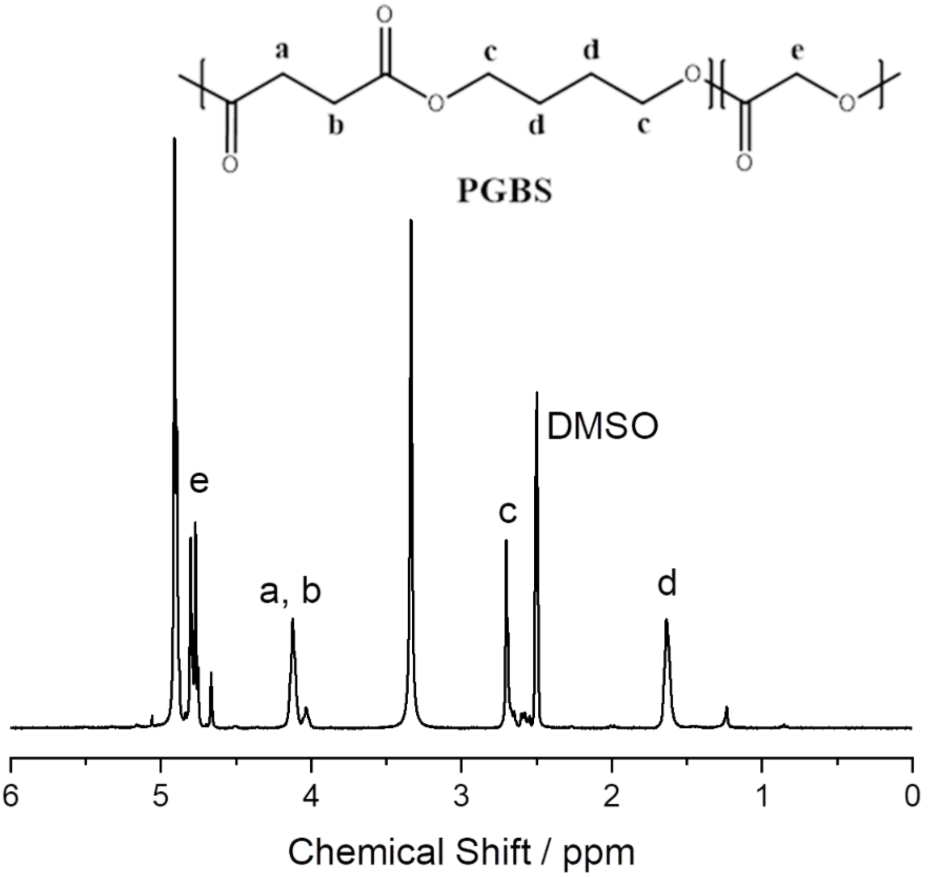

Figure S7. ^{1}H NMR (400 MHz) spectrum of PGBS90 copolymer in DMSO-d_6.

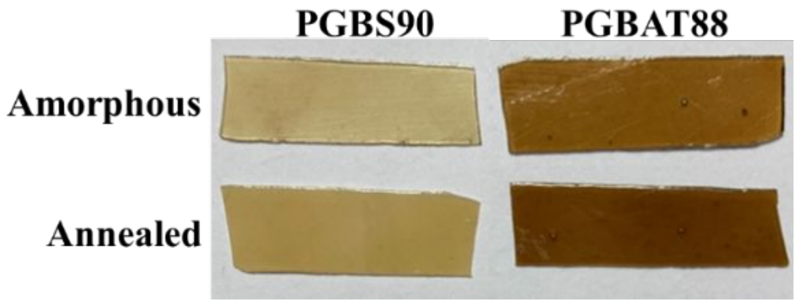

Figure S8. Appearances of amorphous and annealed films of PGBS90 and PGBAT88.

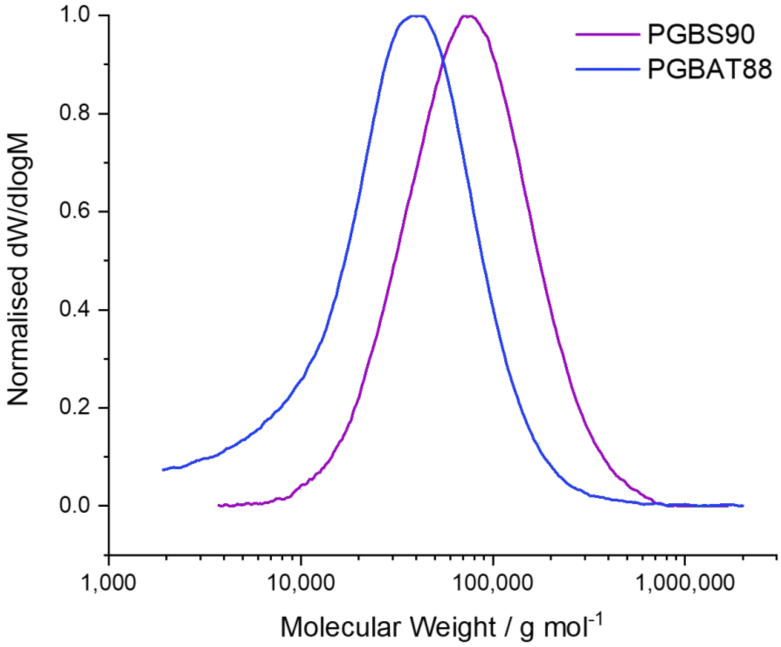

Figure S9. SEC traces of PGBS90 and PGBAT88 in DMF as eluent.
